# Genetic Diversity of Siberian Isolates of *Borrelia burgdorferi* Sensu Lato by the *osp*A Gene

**DOI:** 10.3390/microorganisms13122825

**Published:** 2025-12-11

**Authors:** Yana Igolkina, Vera Rar, Valeriy Yakimenko, Alevtina Bardasheva, Valeria Fedorets, Alfrid Karimov, Gavril Rubtsov, Tamara Epikhina, Nina Tikunova

**Affiliations:** 1 Institute of Chemical Biology and Fundamental Medicine SB RAS, 630090 Novosibirsk, Russia; igolkina@inbox.ru (Y.I.); herba12@mail.ru (A.B.); v.fedorets@alumni.nsu.ru (V.F.); tikunova@1bio.ru (N.T.); 2 Omsk Research Institute of Natural Foci Infections, 644080 Omsk, Russia; vyakimenko78@yandex.ru (V.Y.);

**Keywords:** “*Candidatus* Borrelia sibirica”, *Borrelia bavariensis*, *Borrelia afzelii*, *Ixodes* spp., *osp*A gene, phylogenetic analysis, in silico typing scheme

## Abstract

The genetic diversity of the *osp*A gene of the *Borrelia burgdorferi* sensu lato species complex encoding outer surface protein A has been widely investigated. However, the information on the genetic variability of *Borrelia* isolates from Siberia for this gene is limited. In this study, we analyzed complete *osp*A gene sequences from 36 *Borrelia* isolates from Western Siberia, comprising 6 *Borrelia afzelii*, 16 *Borrelia bavariensis*, 1 *Borrelia garinii*, and 13 “*Candidatus* Borrelia sibirica” isolates. The obtained *osp*A gene sequences of *B. afzelii* were conserved and formed a single clade. In contrast, *B. bavariensis* sequences were highly variable, segregating into two distinct clades consistent with the phylogeography of Asian isolates. Notably, the *B. bavariensis* samples identified in molted *Ixodes trianguliceps* and *Ixodes apronophorus* were first characterized for the *osp*A gene; the obtained sequences corresponded to those from *I. persulcatus*. This study provides the first characterization of the *osp*A gene in “*Candidatus* B. sibirica”, revealing highly conserved sequences (99.8–100% intraspecific identity). The *osp*A gene sequences of “*Candidatus* B. sibirica” shared less than 88.7% identity with those of other *Borrelia* genospecies. Phylogenetic analysis placed “*Candidatus* B. sibirica” in a unique, well-supported clade, confirming its distinct phylogenetic status and suggesting potential ecological specialization in nidicolous *Ixodes* species.

## 1. Introduction

Spirochetes of the genus *Borrelia*, the causative agents of vector-borne diseases, namely, Lyme borreliosis (LB) and relapsing fever, are transmitted to humans and animals through the bites of ticks and lice. The *Borrelia burgdorferi* sensu lato (s.l.) complex comprises a group of tick-transmitted pathogens, some of which are causative agents of LB. This group includes more than 20 accepted and proposed or candidate *Borrelia* genospecies, at least 9 of which are known to infect humans [1,2]. *Borrelia burgdorferi* sensu stricto (s.s.), *Borrelia afzelii*, *Borrelia garinii*, and *Borrelia bavariensis* are the main etiological agents of LB in the temperate zones of the Northern Hemisphere [3].

The distribution of these genospecies varies geographically. In North America, *B. burgdorferi* s.s is the main causative agent of Lyme disease, whereas in Europe, most human cases are attributed to *B. afzelii*, *B. garinii*, and *B. bavariensis* and, less frequently, to *B. burgdorferi* s.s. [4]. Certain genospecies have been shown to be more frequently associated with specific symptoms, including arthritis (*B. burgdorferi* s.s.), neuroborreliosis (*B. garinii* and *B. bavariensis*), acrodermatitis chronica atrophicans, and borrelial lymphocytoma (*B. afzelii*) [5,6,7,8].

Four known species of the *B. burgdorferi* s.l. complex have been found in Siberia, including the widespread *B. afzelii*, *B. bavariensis*, and *B. garinii*, as well as rarely found *B. valaisiana* [9,10,11,12,13]. Of these, *B. garinii* is predominantly detected in *Ixodes pavlovskyi*, while *B. bavariensis* and *B. afzelii* are prevalent in *Ixodes persulcatus* [10,13]. Regarding less studied *Ixodes* ticks, molted *Ixodes apronophorus* and *Ixodes trianguliceps* in Siberia have been shown to be infected with *B. bavariensis* [12], while both *B. afzelii* and *B. bavariensis* strains have been isolated from feeding *I. trianguliceps* in the Urals [14].

Recently, a proposed new genospecies, “*Candidatus* Borrelia sibirica”, was detected in small mammals, in *Ixodes* spp. ticks feeding on small mammals, and in a single molted *I. apronophorus* at several locations within *I. apronophorus* distribution areas in Western Siberia [12]. This candidate genospecies was genetically characterized by a single-locus analysis of the *clp*A, *p*83/100, and 16S rRNA genes and the 5S-23S rRNA intergenic spacer (IGS) and by multi-locus sequence analysis of concatenated sequences of eight housekeeping genes [15]. Phylogenetic analyses revealed that “*Candidatus* B. sibirica” is highly conserved across all examined genetic loci and forms distinct, separate clades [12]. Notably, “*Candidatus* B. sibirica” is most genetically similar to *B. garinii* and *B. bavariensis*.

All studied *B. burgdorferi* s.l. genospecies produce outer surface protein A (OspA) [16]. This integral surface protein is abundantly expressed by *Borrelia* spp. within the midguts of unfed ticks but downregulated during blood feeding [17]. The *osp*A gene is located on linear plasmid 54 (lp54) [18]. Its open reading frame typically ranges from 819 to 825 base pairs in length, encoding a protein of approximately 30 kDa [16]. Initially, *B. burgdoferi* s.l. spirochetes were divided into eight serotypes based on their reactivity to anti-OspA monoclonal antibodies [19,20]. OspA serotypes generally correlate with specific genospecies: *B. burgdorferi* s.s. is typed as serotype 1; *B. afzelii* and *B. bavariensis* are typed as serotypes 2 and 4, respectively; and *B. garinii*, the most heterogeneous species among the *B. burgdorferi* s.l. complex, is typed as serotypes 3, 5, 6, 7, and 8. Recently, Lee and co-authors [21], based on analysis of 90 unique OspA protein variants, proposed a sequence-based OspA in silico typing (IST) scheme. This IST typing includes IST1–IST8, which correspond to OpsA serotypes 1–8, previously identified by traditional serological methods, and potentially novel OspA types corresponding to OspA variants of *B. bavariensis* from Asia (IST9, IST10), *B. garinii* (IST11, IST12), and other *Borrelia* genospecies (IST13–IST17). The *osp*A gene exhibits significant diversity, not only across different *Borrelia* genospecies but also among strains within a single species. Notably, different ISTs within a single genospecies do not always cluster together. Thus, *B. bavariensis* strains do not form a monophyletic group but are divided into three clades, corresponding to three different ISTs. These include the highly conserved IST4, identified in the *I. ricinus* distribution area, and the more variable IST9 and IST10, which are found in Asia [21].

The genetic diversity of different *Borrelia* genospecies for the *osp*A gene has been investigated in a number of studies [16,21,22,23,24,25,26]. However, data on the *osp*A variability of Siberian *Borrelia* isolates remain limited and primarily include strains isolated from *I. persulcatus* and *I. pavlovskyi* collected in the Tomsk and Novosibirsk surroundings [27].

This study aimed to investigate the genetic diversity of the recently identified species “*Candidatus* B. sibirica” from Omsk province for the *osp*A gene and compare it with the *osp*A gene diversity of other *B. burgdorferi* s.l. isolates from the same locations. Particular attention was paid to isolates from the poorly studied tick species *I. trianguliceps* and *I. apronophorus*.

## 2. Materials and Methods

### 2.1. Sampling

All experiments with animals were approved by the Animal Welfare Act of the Omsk Research Institute of Natural Foci Infections, according to the guidelines for experiments with laboratory animals (Supplement to the Order of the Russian Ministry of Health, no. 755, of 12 August 1977). The study was approved by the Bioethical Committee of the Omsk Research Institute of Natural Foci Infections (Protocol No. 4, 17 February 2016; Protocol No.1, 15 March 2024).

**Figure 1:**
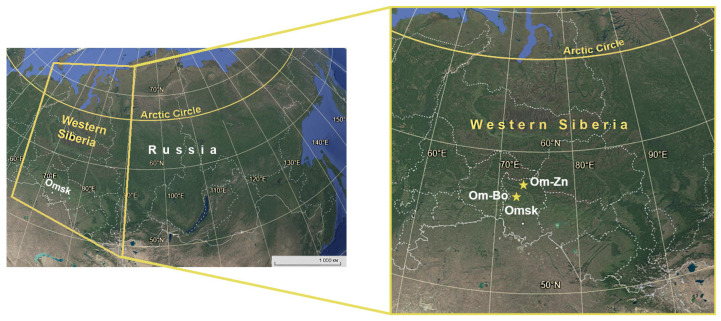
Map showing the location of sampling sites. Sampling sites are marked by stars.

Sampling was conducted in 2016, 2024, and 2025 at two locations of approximately 20 km^2^ each within the forest zone of Omsk province, Western Siberia; the sampling locations and tick collection from small mammals have been previously described [12]. Briefly, the first site (Om-Bo) was located in Bolsheukov district (56°46′ N, 72°03′ E), and the second site (Om-Zn) was situated in Znamenskiy district (57°23′ N, 73°40′ E) (Figure 1). Small mammals were captured using live traps, and attached *Ixodes* spp. ticks were removed by forceps. Some engorged larvae were transported to the laboratory and allowed to molt into nymphs, while other ticks were frozen and stored at −70 °C until DNA extraction. In addition, unfed adult *I. persulcatus* specimens were collected by flagging from vegetation. Identification of questing ticks was carried out using a stereomicroscope, MC-800 (Micros, Sankt Veit an der Glan, Austria), according to morphological criteria [28]. To identify ticks collected from small mammals, multiplex PCR for the ITS2 locus with primers specific to *I. persulcatus*, *I. trianguliceps*, and *I. apronophorus* (Table 1) was conducted, as previously described [29].

Some of the molted ticks and ticks from vegetation were stored at 6–10 °C and used for cultivation of *B. burgdorferi* s.l. spirochetes in BSK-H medium. The remaining ticks were frozen and stored at −70 °C until DNA extraction.

### 2.2. Borrelia Cultivation

Ticks were washed sequentially in 4% H_2_O_2_ for 2 min, followed by two washes in 70% ethanol and two washes in sterile phosphate-buffered saline (PBS), for 5 min each. The ticks were then homogenized using sterile pestles in 80 µL of BSK-H medium. The resulting suspensions were centrifuged at 100 g for 30 s; 40 µL of each supernatant was used to cultivate the spirochetes; the remaining tick material was used for DNA extraction. *Borrelia* was cultured in BSK-H medium (Sigma Chemical Co., St. Louis, Mo, USA) supplemented with 6% rabbit serum (Sigma, USA) and containing 1× antibiotic mixture (HiMedia, Mumbai, India) in tightly sealed 0.6 mL tubes at 33 °C without agitation. The volume of initial cultures was 570 µL. Spirochete growth was monitored for 1–3 months under a microscope at 400× magnification (Axio Imager A1, Zeiss, Jena, Germany). To obtain stable isolates, the primary isolates were subcultured into a fresh culture medium within 1.5 mL screw cap tubes at a final working volume of 1.5 mL. From each obtained isolate, 30 µL of culture medium was collected and used for DNA extraction.

### 2.3. DNA Extraction

To prevent cross-contamination, DNA extraction, PCR assays, and electrophoresis were conducted in separate rooms. Total DNA was extracted from whole frozen ticks, from tick suspensions obtained during *Borrelia* cultivation, and from culture medium. Before DNA extraction, ticks were washed individually with bidistilled water, then with 70% ethanol, and finally with bidistilled water for 5 min each. Ticks were homogenized with a MagNA Lyser system (Roche Diagnostics, Basel, Switzerland). DNA extraction was conducted using the Proba NK kit (DNA-Technology, Moscow, Russia), according to the manufacturer’s instructions. DNA was eluted in 50 μL of elution buffer and stored at −70 °C.

### 2.4. Borrelia burgdorferi s.l. Detection, Genotyping, and Characterization of the ospA Gene

*Borrelia burgdorferi* s.l. DNA was detected by real-time PCR using a RealBest DNA *Borrelia burgdorferi* s.l. kit (Vector-Best, Novosibirsk, Russia). For all positive samples, the fragments of IGS, *clp*A, and/or *p*83/100 were amplified using nested PCR with primers specified in Table 1. The *B. burgdorferi* s.l. species were determined by sequencing *clp*A or IGS fragments and by comparing the lengths of the obtained PCR fragments of the *p*83/100 gene (336 bp for *B. afzelii* and 426 bp for *B. bavariensis*), as described previously [10]. “*Candidatus* B. sibirica” was identified by nested reactions with species-specific primers for the IGS region (Table 1). For a number of positive samples belonging to different genospecies, the *osp*A gene fragments were amplified using nested PCR with the primers indicated in Table 1 and used for subsequent sequencing.

### 2.5. Phylogenetic Analysis

The obtained PCR fragments were purified in 0.6% SeaKem^®^ GTG-agarose (Lonza, Haifa, Israel). Sanger sequencing was conducted with primers indicated in Table 1 in both directions using a BigDye Terminator V. 3.1 Cycling Sequencing Kit (Applied Biosystems, Carlsbad, CA, USA). Sanger reaction products were analyzed using an ABI 3500 Genetic Analyzer (Applied Biosystems, Carlsbad, CA, USA). The quality of chromatograms was assessed by Phred quality score using the uGene v. 52.1 software [30]. The determined *clp*A and IGS sequences were compared with those from the NCBI website using BLASTN (https://blast.ncbi.nlm.nih.gov/Blast.cgi, accessed on 15 October 2025). The determined *clp*A gene sequences were also analyzed using the PubMLST website (https://pubmlst.org/organisms/borrelia-spp, accessed on 15 October 2025). Phylogenetic analysis was performed using the maximum likelihood (ML) method based on the General Time Reversible with Gamma distribution (GTR + G) model. The best-fitting substitution model was determined with the Bayesian Information Criterion (BIC) using the ML model test implemented in MEGA 7.0 [31]. Nucleotide diversity (π) and haplotype diversity (Hd) were computed for each group using standard population-genetic estimators implemented in the ape and pegas R packages (R v4.4.2; ape v5.8; pegas v1.2) [32]. Statistical comparisons between groups were performed in R with appropriate multiple-testing correction (Benjamini–Hochberg FDR). *p* < 0.05 was regarded as significant.

### 2.6. Nucleotide Sequence Accession Numbers

Nucleotide sequences determined in this study were submitted to the GenBank database (http://www.ncbi.nlm.nih.gov, accessed on 15 November 2025) under accession numbers PX461036-PX461050 and PX513318-PX513325.

## 3. Results

### 3.1. Borrelia burgdorferi s.l. Detection and Species Determination

Based on the results of PCR detection, *B. burgdorferi* s.l. DNA was found in 32/123 (26.0%) *I. persulcatus* samples collected from vegetation in 2024–2025; in 127/261 (48.7%) *Ixodes* spp. (121/232 *I. persulcatus*, 3/16 *I. trianguliceps,* and 3/13 *I. apronophorus*) collected from rodents in 2024–2025 as larvae and molted into nymphs; and in 5/44 (11.4%) *Ixodes* spp. (5/10 *I. apronophorus*) collected from rodents in 2016 (Table 2). Stable *Borrelia* strains or primary isolates, confirmed by microscopy, were obtained from ten PCR-positive ticks collected from vegetation and from two molted ticks: one *I. persulcatus* and one *I. trianguliceps*. Genotyping of the *B. burgdorferi* s.l. isolates revealed two genospecies—*B. bavariensis* and *B. afzelii*—in ticks collected from both vegetation and rodents in 2024 and 2025, with *B. bavariensis* predominating in both locations. In contrast, only “*Candidatus* B. sibirica” was identified in ticks collected from rodents at the Om-Bo site in 2016 (Table 2).

### 3.2. Results of ospA Characterization and Phylogenetic Analysis

Complete *osp*A gene sequences were determined for 36 samples, comprising 13 “*Candidatus* B. sibirica”, 6 *B. afzelii,* 1 *B. garinii*, and 16 *B. bavariensis* isolates, which were identified in *Ixodes* spp. ticks and a blood sample from *Sorex araneus* from different locations (Table 3).

Twelve of the “*Candidatus* B. sibirica” samples were detected in *Ixode*s spp. collected from rodents at the Om-Bo site in 2016. This subset included three new specimens identified in this study and nine isolates that were previously described [12]. The remaining “*Candidatus* B. sibirica” sample was identified in the blood of *S. araneus* captured in 2024 at the Om-Zn site [33] (Table 3). Of the six genotyped *B. afzelii* samples, five samples were identified directly in *I. persulcatus* from both locations, and one sample was from a strain isolated from *I. persulcatus*. The sixteen *B. bavariensis* specimens were identified from the following sources: six samples from cultured strains from *I. persulcatus* (n = 5) and *I. trianguliceps* (n = 1); three samples from questing *I. persulcatus* (n = 3); and seven samples from molted ticks, including *I. persulcatus* (n = 4), *I. apronophorus* (n = 2), and *I. trianguliceps* (n = 1) (Table 3). In addition, one *B. garinii* strain isolated from *I. pavlovskyi* from Novosibirsk Province was genotyped for the *osp*A gene (Table 3).

**Figure 2:**
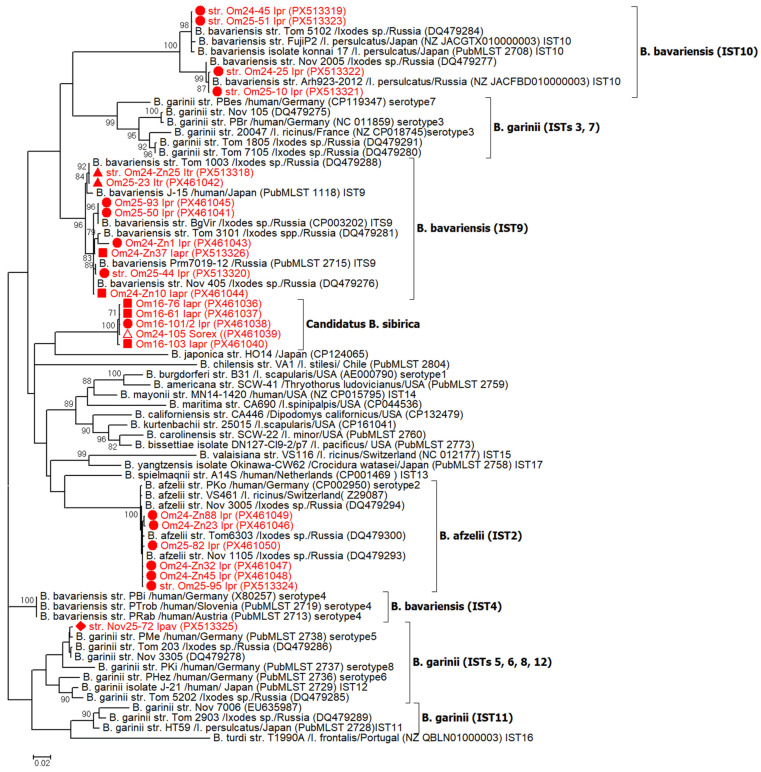
Phylogenetic tree constructed by the ML method based on sequences of *osp*A gene fragments (length, 819–825 bp) of *B. burgdorferi* s.l. Sequences obtained in this study are marked in red. ●—*I. persulcatus*; ■—*I. apronophorus; ▲*—*I. trianguliceps;* ♦—*I. pavlovskyi*; Δ—a specimen from a small mammal. The scale-bar indicates an evolutionary distance of 0.02 nucleotides per position. Significant bootstrap values (*>*70%) are shown on the nodes.

The results of *B. burgdorferi* s.l. species determination for the *osp*A gene were consistent with the results obtained from analyses of the *clp*A, *p*83/10*0*, and IGS sequences. The *osp*A gene sequences of “*Candidatus* B. sibirica” were determined for the first time in this study. These sequences were highly conserved, with 12 of the 13 being identical. A single sequence (PX461040) differed by two nucleotide substitutions, one of which was synonymous, resulting in an intraspecific identity of 99.8–100%. The *osp*A gene sequences of “*Candidatus* B. sibirica” were genetically distant from the corresponding sequences of other *Borrelia* genospecies, showing the level of nucleotide identity varying from 81.5% (*B. valaisiana*, IST15) to 88.7% (*B. bavariensis*, IST4) (Table 4). Similarly, the deduced OspA amino acid sequences shared identities ranging from 69.2% (*B. valaisiana*) to 84.2% (*B. yangtzensis*). On the phylogenetic tree, the *osp*A gene sequences of “*Candidatus* B. sibirica” with a high level of support formed a separate branch, demonstrating its distinct phylogenetic status (Figure 2).

The obtained *B. afzelii osp*A gene sequences were also highly conserved. Five distinct sequence variants of the *osp*A gene were found. One variant was identical to a known sequence of *B. afzelii* strain, Nov1105 (DQ479293), while the other four variants differed from this sequence by 1–2 nucleotide substitutions and from each other by 1–3 substitutions, corresponding to a 99.6–99.9% identity level. The determined *osp*A sequences of *B. afzelii*, together with the known *B. afzelii* sequences, formed a single group on the phylogenetic tree, belonging to the IST2 cluster (Figure 2).

In contrast to the highly conserved sequences of “*Candidatus* B. sibirica” and *B. afzelii*, the determined *osp*A sequences of *B. bavariensis* exhibited considerable diversity. These sequences differed from each other by 1–128 mismatches, including six indels, demonstrating 84.4–99.9% identity. A total of eight different sequence variants were identified. Five of these variants were identical to known sequences of the strains BgVir (DQ479279), Nov 405 (DQ479276), Tom 1003 (DQ479288), Tom 5102 (DQ479284), and Arh923-2012 (NZ_JACFBD010000003), while the remaining three variants were unique and differed from the known sequences by 1–11 substitutions. Notably, 4 of the 16 *osp*A sequences of *B. bavariensis* (1 from *I. trianguliceps* and 3 from *I. persulcatus*) contained multiple polymorphic sites (ranging from 12 to over 50 single-nucleotide polymorphisms (SNPs)) and were consequently excluded from the phylogenetic analysis. The remaining sequences belonged to two well-supported clusters. The first cluster comprised known sequences corresponding to IST9 (e.g., strains BgVir and J-15) and eight novel sequences from this study, identified in various tick species from both the Om-Bo and Om-Zn sites. The second cluster consisted of sequences corresponding to IST10 (e.g., strains Fujip2 and Arh923-2012) and four novel sequences from this study, all of which were derived from *I. persulcatus* collected at the Om-Bo site (Figure 2, Table 3).

The sequences from the cluster IST9 obtained in this study differed from each other by 6–25 mismatches (including three indels), corresponding to a sequence identity of 96.9–99.3%, whereas sequences from the cluster IST10 differed from each other by 1–39 mismatches (including three indels), representing a 95.2–99.9% identity range. This study also reports the first determination of *B. bavariensis osp*A sequences from samples identified in *I. trianguliceps* and *I. apronophorus*. Notably, these sequences were not unique to these tick species. They were either identical to sequences previously found in *I. persulcatus* or, as in the case of one sample from *I. apronophorus* (PX513326), differed from them by five SNPs.

In addition to *B. burgdorferi* s.l. strains from Omsk province, the obtained *osp*A gene sequence of *B. garinii* (strain 72) isolated from *I. pavlovskyi* in Novosibirsk province was included in the phylogenetic analysis. The determined *B. garinii* sequence differed from the closest sequence (strain Tom 203, DQ479286) by three nucleotide substitutions and clustered with *B. garinii* sequences corresponding to IST5 (e.g., strain PMe, Germany) (Figure 2).

In total, the *Borrelia* isolates identified in Omsk province represent four distinct genospecies based on nuclear loci. Analysis of their *osp*A plasmid locus classifies these isolates into four separate ospA-based serotype clusters.

Analysis of *ospA* sequences from different *Borrelia* genospecies—including those obtained in this study and previously published sequences from Western Siberia—revealed that “*Candidatus* B. sibirica” exhibits significantly lower genetic diversity than other genospecies. Specifically, it has the lowest nucleotide (π = 0.0004) and haplotype (Hd = 0.154) diversity. The differences in nucleotide diversity were statistically significant (*p* < 0.05) between “*Candidatus* B. sibirica” and *B. bavariensis* from the IST9 and IST10 clusters but not between “*Candidatus* B. sibirica” and *B. afzelii*. The differences in haplotype diversity were also significant (*p* < 0.05) between “*Candidatus* B. sibirica” and all other IST clusters (Table 5).

Comparisons among other genospecies revealed that *B. afzelii* had lower nucleotide diversity (π = 0.0015) than *B. bavariensis* from both the IST9 (π = 0.0150) and IST10 (π = 0.0244) clusters, and this difference was statistically significant (*p* < 0.05). Haplotype diversity was significantly higher (*p* < 0.05) for *B. bavariensis* of the IST9 cluster (Hd = 0.872) than for the IST10 cluster (Hd = 0.733) and *B. afzelii* (Hd = 0.75) (Table 5). As expected, pooled *B. bavariensis* isolates from both clusters exhibited significantly higher (*p* < 0.05) nucleotide (π = 0.0785) and haplotype (Hd = 0.913) diversity compared to the individual IST clusters analyzed separately (Table 5).

## 4. Discussion

*Borrelia* OspA protein is crucial for bacterial survival in ticks, as it is essential for spirochete adhesion to the tick midgut and further colonization [34,35]. Due to its important role in transmission, OspA is a well-known target for LB vaccines, as antibodies against OspA can prevent transmission of bacteria from infected ticks to hosts. Consequently, the study of *osp*A gene diversity is important for the development of multivalent OspA-based vaccines directed against a wide range of *Borrelia* genospecies. Eight OspA serotypes of *B. burgdorferi* s.l. are known; in addition, nine potential serotypes have recently been identified based on OspA sequence typing [21].

This study provides the first data on the diversity of the *osp*A gene in *B. burgdorferi* s.l. isolates from the sympatric areas of *I. persulcatus*, *I. trianguliceps*, and *I. apronophorus* in Western Siberia. Particular attention is paid to characterizing *ospA* diversity in the recently found species “*Candidatus* B. sibirica,” as well as in isolates derived from the poorly studied nidicolous ticks *I. trianguliceps* and *I. apronophorus.*

The first *osp*A gene sequences were obtained for 13 “*Candidatus* B. sibirica” isolates, which were found in *Ixodes* spp. collected from rodents at site Om-Bo in 2016 and in the blood sample of a shrew at site Om-Zn in 2024 (Table 3). Although the examined samples varied in sampling location, collection year, and type of sample (ticks and blood samples), the obtained *osp*A gene sequences were highly conserved and demonstrated the lowest genotype and haplotype diversity (Table 5). On the phylogenetic tree, these sequences form a separate, well-supported clade and can be considered a potential new IST. The high genetic stability of “*Candidatus* B. sibirica” was previously demonstrated by an analysis of the genomic housekeeping *clp*A gene, the *p*83/100 gene that encodes the outer membrane protein, as well as the ribosomal 16S rRNA gene and 5S-23S IGS for 22 “*Candidatus* B. sibirica” isolates. Moreover, MLST analysis revealed an identical sequence type (ST806) for two isolates from geographically distant regions of Siberia (Altai and Omsk provinces) [12]. The observed high genetic stability at plasmid and nuclear loci may result from the adaptation of “*Candidatu*s B. sibirica” to a narrow ecological niche due to its putative association with the scarce tick *I. apronophorus.*

The observed diversity of Siberian isolates of *B. afzelii* and *B. bavariensis* for the *osp*A gene is consistent with the data from previous publications [21,23,25,27]. The determined *B. afzelii* sequences were conserved and, together with other known *B. afzelii* sequences from Europe and Asia, formed a single well-supported clade, while *B. bavariensis* sequences were variable and subdivided into two distant clades corresponding to IST9 and IST10, widely spread in Asia (Figure 2).

For nidicolous *I. trianguliceps* and *I. apronophorus* ticks, whose life stages feed primarily on small mammals, examining engorged ticks collected from hosts and subsequently molted in the laboratory can provide crucial insights into specific tick–pathogen associations. In this study, *Borrelia* isolates from molted *I. trianguliceps* and *I. apronophorus* were genotyped for the *osp*A gene for the first time. Only *B. bavariensis* was found in these ticks, confirming our previous findings [12]. Notably, the obtained *B. bavariensis* sequences from *I. trianguliceps* and *I. apronophorus* were identical to the *osp*A sequences identified in *I. persulcatus* from Siberia. Previous genotyping of *B. bavariensis* isolates from molted *I. trianguliceps* and *I. apronophorus* for the *clp*A and *p*83/100 genes also failed to reveal any tick-specific variants [12]. Taken together, these results indicate that nidicolous ticks, along with human-biting *I. persulcatus*, may participate in common enzootic cycles associated with highly pathogenic species *B. bavariensis*.

*Ixodes apronophorus* is widely distributed across Europe and Western Siberia, inhabiting sympatric areas with *I. ricinus* in Europe and *I. persulcatus* in Asia [36,37]. However, because of mosaic distribution and a generally low abundance, this tick species remains poorly studied. To date, *Borrelia* in *I. apronophorus* has only been found in ticks in the Omsk province in this and previous studies. Two *Borrelia* species are suggested to be associated with *I. apronophorus* [12]. Of these, *B. bavariensis* isolates identified in *I. apronophorus* are genetically variable, while “*Candidatus* B. sibirica” appears to be the most genetically conserved species in the studied locations. A similar pattern of genetic stability has been observed in the European genotype of *B. bavariensis* (OspA serotype 4). Genetic and genomic data indicate that *B. bavariensis* spread from Asia to Europe and experienced a significant genetic bottleneck during this invasion. This bottleneck was likely driven by its adaptation to a new vector, shifting from *I. persulcatus* to *I. ricinus* [38,39]. We hypothesize that a similar population bottleneck may have accompanied the adaptation of “*Candidatus* B. sibirica” to *I. apronophorus*. Thus, “*Candidatus* B. sibirica” may have evolved from a closely related *B. bavariensis* or *B. garinii* species with a vector switch from *I. ricinus* or *I. persulcatus* to *I. apronophorus*. Notably, further studies of *Borrelia* in *I. apronophorus* from regions where it is sympatric with *I. ricinus* could elucidate both the broader geographical distribution and genetic diversity of “*Candidatus* B. sibirica”, as well as the evolutionary events that led to its emergence.

## Figures and Tables

**Table 1:** Primers used for identification and genotyping of *Ixodes* spp. and *B. burgdorferi* s.l.

Locus	Organism	Reaction	Primer Name	Primer Sequences 5′-3′	T ^#^ (°C)	Size (bp)	References
ITS2	*Ixodes* spp.	Multiplex	dITS29	ccttcccgtggcttcgtctgt	60		[29]
ITS2	*I. persulcatus*		IP_rev	ctgtacatccgtccatttaggc		412–413	
ITS2	*I. trianguliceps*		IT_rev	cggcaatcgaacgacgt		222	
ITS2	*I. apronophorus*		IA_rev	tggcgaagatcatttgagttg		706–709	
IGS	*B. burgdorferi* s.l.	Primary	NC1	cctgttatcattccgaacacag	50	350–384	[10]
			NC2	tactccattcggtaatcttggg			
		Nested	NC3	tactgcgagttcgcgggag	50	237–271	
			NC4	cctaggcattcaccatagac			
	“*Ca.* B. sibirica”	Nested	Bsib	ataaaacattctaaaaaaatgaaca	50	172	This study
			NC4	cctaggcattcaccatagac			[10]
*clp*A	*B. burgdorferi* s.l.	Primary	clpAF1237	aaagatagatttcttccagac	50	982	[15]
			clpAR2218	gaatttcatctattaaaagctttc			
		Nested	clpAF1255	gacaaagcttttgatattttag	50	850	
			clpAR2104	caaaaaaaacatcaaattttctatctc			
*p*83/100	*B. burgdorferi* s.l.	Primary	F7	ttcaaagggatactgttagagag	50	438–528	[10]
			F10	aagaaggcttatctaatggtgatg			
		Nested	F5	acctggtgatgtaagttctcc	54	336–426	
			F12	ctaacctcattgttgttagactt			
*osp*A	*B. burgdorferi* s.l.	Primary	pA3	ctatttgttatttgttaatcttatac	48	935–944	[27]
			pA4	gcaaatcctagtaaatattgtttc			
		Nested	ospA91F	aaaaggagaatatattatgaaaa	48	855–862	This study
			ospA924R	tattktttcataaattctcctt			
*osp*A	*B. burgdorferi* s.l.	Sequencing	ospA561F *	ccggaaaagctaaagaagtt			This study
			ospA561R *	aacttctttagcttttccgg			

* Primers used only for sequencing. # Annealing temperature.

**Table 2:** Results of *Borrelia burgdorferi* s.l. detection.

Site	Sampling Period	Source of Isolation	No. of Ticks	No. (%) of Ticks Containing Bbsl DNA	Results of Bbsl Species Determination				Samples Used for *osp*A Genotyping		
					*B.a*	*B.bav*	*Ca.*B.sib	Mixed	*B.a*	*B.bav*	*Ca.*B.sib
Om-Bo	June 2024	*I. persulcatus*, flagging	28	8 (28.6)	2	5	0	1	0	1	0
Om-Bo	May 2025	*I. persulcatus*, flagging	95	24 (25.3)	8	9	0	7	2	6	0
Om-Zn	September 2024	*Ixodes* spp, from rodents, molted:	90	28 (31.1)	5	20	0	3	4	5	0
		*I. apronophorus*	8	3 (37.5)	0	3	0	0	0	2	0
		*I. trianguliceps*	14	2 (14.3)	0	2	0	0	0	2	0
		*I. persulcatus*	68	23 (33.8)	5	15	0	3	4	1	0
Om-Bo	September 2024	*Ixodes* spp, from rodents, molted:	69	41 (59.4)	11	13	0	17	0	1	0
		*I. persulcatus*	69	41 (59.4)	11	13	0	17	0	1	0
Om-Bo	June 2025	*Ixodes* spp, from rodents, molted:	102	58 (56.8)	4	41	0	13	0	3	0
		*I. apronophorus*	5	0	0	0	0	0	0	0	0
		*I. trianguliceps*	2	1	0	1	0	0	0	1	0
		*I. persulcatus*	95	57 (60.0)	4	40	0	13	0	2	0
Om-Bo	June 2016	*Ixodes* spp. from rodents:	44	5 (11.4)	0	0	5	0	0	0	3
		*I. apronophorus*	10	5 (50.0)	0	0	5	0	0	0	3
		*I. trianguliceps*	5	0	0	0	0	0	0	0	0
		*I. persulcatus*	29	0	0	0	0	0	0	0	0

Abbr. Bbsl—*B. burgdorferi* s.l., *B.a*—*B. afzelii*, *B.bav*—*B. bavariensis*; *Ca.*B.sib—“*Candidatus* B. sibirica”; mixed—*B. afzelii* and *B. bavariensis* mixed infection.

**Table 3:** Results of *B. burgdorferi* s.l. genotyping for the *osp*A gene.

Isolate	Site	Source of Isolation	Results of *Borrelia* Genotyping by		Cluster by *osp*A Gene	GenBank Accession No.
			*clp*A	*osp*A		
Om16-76_Iapr	Om-Bo	*I.apr*, F; from *A. agrarius*	*Ca.*B.sib	*Ca.*B.sib	new	PX461036
Om16-61_Iapr	Om-Bo	*I.apr*, N; from *C. glareolus*	*Ca.*B.sib	*Ca.*B.sib	new	PX461037
Om16-101/2-Ipr *	Om-Bo	*I.pers*, N; from *C. glareolus*	*Ca.*B.sib	*Ca.*B.sib	new	PX461038
Om16-103-Iapr *	Om-Bo	*I.apr*, F; from *Ar. amphibious*	*Ca.*B.sib	*Ca.*B.sib	new	PX461040
Om16-75-Iapr *	Om-Bo	*I.apr*, N; from *C. glareolus*	*Ca.*B.sib	*Ca.*B.sib	new	
Om16-79/2-Iapr *	Om-Bo	*I.apr*; F; from *M. oeconomus*	*Ca.*B.sib	*Ca.*B.sib	new	
Om16-101/1-Ipr *	Om-Bo	*I.pers*, L; from *C. glareolus*	*Ca.*B.sib	*Ca.*B.sib	new	
Om16-132/2-Ipr *	Om-Bo	*I.pers,* L; from *C. glareolus*	*Ca.*B.sib	*Ca.*B.sib	new	
Om16-132/4-Ipr *	Om-Bo	*I.pers,* L; from *C. glareolus*	*Ca.*B.sib	*Ca.*B.sib	new	
Om16-132/5-Ipr *	Om-Bo	*I.pers,* L; from *C. glareolus*	*Ca.*B.sib	*Ca.*B.sib	new	
Om16-132/7-Ipr *	Om-Bo	*I pers,* L; from *C. glareolus*	*Ca.*B.sib	*Ca.*B.sib	new	
Om16-147_Iapr	Om-Bo	*I.apr*, F; from *Ar. amphibious*	*Ca.*B.sib	*Ca.*B.sib	new	
Om24-105_Sorex **	Om-Zn	*Sorex araneus*, blood	*Ca.*B.sib	*Ca.*B.sib	new	PX461039
Om25-93_Ipr	Om-Bo	*I.pers,* F; flag	*B.bav*	*B.bav*	IST9	PX461045
Om24-Zn1_Ipr	Om-Zn	*I.pers,* L molted into N; from *C. rutilus*	*B.bav*	*B.bav*	IST9	PX461043
Om24-Zn10_Iapr	Om-Zn	*I.apr,* L molted into N; from *C. rutilus*	*B.bav*	*B.bav*	IST9	PX461044
Om24-Zn37_Iapr	Om-Zn	*I.apr,* L molted into N; from *C. rutilus*	*B.bav*	*B.bav*	IST9	PX513326
Om24-Zn43_Itr	Om-Zn	*I.tr,* L molted into N; from *C. rutilus*	*B.bav*	mixed	n.d.	
Om25-23_Itr	Om-Bo	*I.tr,* L molted into N; from *M. oeconomys*	*B.bav*	*B.bav*	IST9	PX461042
Om25-50_Ipr	Om-Bo	*I.pers,* L molted into N; from *A. agrarius*	*B.bav*	*B.bav*	IST9	PX461041
Om25-64_Ipr	Om-Bo	*I.pers,* L molted into N; from *C. rutilus*	*B.bav*	mixed	n.d.	
Om25 34_Ipr	Om-Bo	*I.pers,* F; flag	*B.bav*	mixed	n.d.	
**Om25 51_Ipr**	Om-Bo	*I.pers,* M; flag	*B.bav*	*B.bav*	IST10	PX513323
**Om25-10_Ipr**	Om-Bo	*I.pers,* F; flag	*B.bav*	*B.bav*	IST10	PX513321
**Om25-44_Ipr**	Om-Bo	*I.pers,* F; flag	*B.bav*	*B.bav*	IST9	PX513320
Om25-73_Ipr	Om-Bo	*I.pers,* F; flag	*B.bav*	mixed	n.d.	
**Om24-Zn_25_Itr**	Om-Zn	*I.tr,* L molted into N; from *C. rutilus*	*B.bav*	*B.bav*	IST9	PX513318
**Om24-45_Ipr**	Om-Bo	*I.pers,* M; flag	*B.bav*	*B.bav*	IST10	PX513319
**Om24-25_Ipr**	Om-Bo	*I.pers,* L molted into N; from *C. rutilus*	*B.bav*	*B.bav*	IST10	PX513322
Om24-Zn23_Ipr	Om-Zn	*I.pers,* L molted into N; from *C. rutilus*	*B. afzelii*	*B. afzelii*	IST2	PX461046
Om24-Zn32_Ipr	Om-Zn	*I.pers,* L molted into N; from *C. rutilus*	*B. afzelii*	*B. afzelii*	IST2	PX461047
Om24-Zn45_Ipr	Om-Zn	*I.pers,* L molted into N; from *C rutilus*	*B. afzelii*	*B. afzelii*	IST2	PX461048
Om24-Zn88_Ipr	Om-Zn	*I.pers,* L molted into N; from *C. rutilus*	*B. afzelii*	*B. afzelii*	IST2	PX461049
Om25-82_Ipr	Om-Bo	*I.pers,* M; flag	*B. afzelii*	*B. afzelii*	IST2	PX461050
**Om25-95_Ipr**	Om-Bo	*I.pers,* F; flag	*B. afzelii*	*B. afzelii*	IST2	PX513324
**Nov25-72_Ipav**	Nov	*I.pavl*; F; flag	*B. garinii*	*B. garinii*	IST5	PX513325

The names of isolates include the year of tick/animal collection–ID_source of isolation. *Borrelia* strains are in boldface. *—isolates were described in [12]; **—an isolate was described in [33]; Abbrs.: n.d.—not determined; L—larva; N—nymph; F—female; M—female; *I.pers*—*I. persulcatus*; *I.tr*—*I. trianguliceps*; *I.apr*—*I. apronophorus*; *I.pavl*—*I. pavlovskyi*; *Ca.*B.sib—“*Candidatus* B. sibirica”; *B.bav*—*B. bavariensis*; *C. rutilus—Clethrionomys rutilus*; *C. glareolus—Clethrionomys rutilus*; *Ar. amphibious*—*Arvicola amphibious*; *A. agrarius*—*Apodemus agrarius*; *M. oeconomus*—*Microtus oeconomus*.

**Table 4:** Identity levels of “*Candidatus* B. sibirica” *osp*A gene nucleotide sequences with those of other *B. burgdorferi* s.l. species.

Species, Strain	Serotype/IST	% Identity
*B. afzelii*, VS461 (Z29087)	2	85.4
***B. bavariensis*, PBi (CP000015)**	**4**	**88.7**
*B. bavariensis* BgVir (CP003202)	9	87.9
*B. bavariensis* FujiP2 (NZ_JACGTX010000003)	10	84.8
*B. burgdorferi*, B31 (AE000790)	1	86.4
*B. garinii*, 20047 (CP028862)	3	84.0
*B. garinii*, PBr (CP001308)	3	84.0
*B. garinii*, PMe (PubMLST 2738)	5	87.2
*B. garinii*, PHez (PubMLST 2736)	6	86.5
*B. garinii*, PBes (CP119347)	7	83.0
*B. garinii*, PKi (PubMLST 2737)	8	85.8
*B. garinii*, HT59 (PubMLST 2728)	11	85.0
*B. garinii*, J-21 (PubMLST 2729)	12	86.8
*B. mayonii* MN14-1420 (NZ_CP015795)	14	87.6
*B. spielmanii* A14S (CP001469)	13	86.8
*B. turdi* Ya501 (AB016975)	16	85.9
*B. valaisiana*, VS116 (CP001433)	15	81.5
*B. yangtzensis* Okinawa-CW62 (PubMLST 2758)	17	87.1

The highest level of identity is marked in bold.

**Table 5:** Summary statistics for intraspecific divergences caused by complete *osp*A gene for *B. burgdorferi* s.l. identified in Western Siberia.

	“*Candidatus* B. sibirica”	*B. afzelii*, IST2	*B. bavariensis*, IST9	*B. bavariensis*, IST10	All *B. bavariensis*
No. of isolates	13	24	13	10	23
Size (bp)	819	822	822	822	822
No. of haplotypes	2	7	7	4	11
No. of polymorphic sites	2	6	37	37	150
Nucleotide diversity, π ± S.D.	0.0004 ± 0.0003	0.0015 ± 0.0002	0.0150 ± 0.0015	0.0244 ± 0.0031	0.0785 ± 0.0056
Haplotype diversity, Hd ± S.D.	0.154 ± 0.126	0.7500 ± 0.073	0.872 ± 0.067	0.733 ± 0.120	0.913 ± 0.033

## Data Availability

The original contributions presented in this study are included in the article. Further inquiries can be directed to the corresponding author.
